# Exploring Use Acceptance of Electric Bicycle-Sharing Systems: An Empirical Study Based on PLS-SEM Analysis

**DOI:** 10.3390/s22187057

**Published:** 2022-09-18

**Authors:** Lijun Pan, Yangkun Xia, Lining Xing, Zhihang Song, Yunbao Xu

**Affiliations:** 1 School of Management, Hunan Institute of Engineering, Xiangtan 411104, China; 2 School of Transportation and Logistics, Central South University of Forestry and Technology, Changsha 410004, China; 3 School of Economics, Hunan Institute of Engineering, Xiangtan 411104, China

**Keywords:** electric bicycle-sharing system, partial least squares, structural equation modeling technology acceptance model

## Abstract

The electric bicycle-sharing system (EBSS) is the fourth-generation urban shared bicycle travel system, which effectively improves the travel efficiency of urban residents and solve the problem of urban congestion. This study attempts to use an extended technology acceptance model (TAM) method to study the acceptance of EBSSs. We had introduced four potential variables, including perceived pleasure (PP), perceived environmental value (PEV), perceived cost (PC), and perceived reliability (PR), into the classic TAM to form a new EBSS-TAM. Data were obtained by using a Likert scale questionnaire from 399 citizens in China. Partial least-squares structural equation modeling (PLS-SEM) with reflective constructs was employed as the analysis method. The results showed that: (1) the EBSS-TAM can explain user behaviors regarding the use of EBSSs. PP has a positive impact on behavior attitude (BA) while having no impact on behavior intention (BI). PEV has no impact on BA and BI. PC has a negative impact on BA and has no impact on BI. PR has a positive impact on BA while having no impact on BI. Perceived ease of use (PEU) has a positive impact on PP and PEV. (2) Younger users (under 35 years old) are more likely to change from liking CBSSs to using EBSSs than older users are. Male users are more satisfied with EBSSs because of their ease of use. The users who never used CBSSs are more likely to perceive the environmental protection value of EBSSs. Some managerial implications were proposed for the EBSSs.

## 1. Introduction

The number of private vehicles across the globe has been steadily increasing. This growth has led to mounting environmental problems, such as noise, traffic congestion, and air pollution [1]. Bike sharing, an environmentally friendly means of transport with health benefits, has received notable attention because of its potential to effectively relieve traffic congestion, mitigate transport-related emissions, and improve public health [2,3,4]. The bike-sharing system was first introduced in Amsterdam in 1965 to meet the demand of ‘last-mile’ travel. Now it is popularized among many cities around the world [5]. According to the Bike-Sharing World Map (accessed on 1 January 2021, https://bikesharingworldmap.com), about 2800 bike-sharing systems have been introduced from 2007 to 2020 all over the world.

Users can access bicycles via one of the following bike-sharing systems: (1) the station-based bike-sharing system (SBBSS), (2) the dockless bike-sharing system (DLBSS), and (3) the electric bike-sharing system (EBSS) [6]. With the development of mobile technology and satellite positioning systems, smart devices and mobile apps are integrated into a bike-sharing system (BSS) to form user-friendly smarter systems that allow users to locate and unlock bikes via smartphone applications. Such systems allow users to park and check out conveniently; thus, many citizens have chosen these systems for middle- and short-range travel.

SBBSSs and DLBSSs are classic bike-sharing systems (CBSS), and these systems utilize traditional motorless bicycles as means for short-range travel. As electric bicycles easily outclass traditional bicycles in terrain adaptability [7], EBSSs have shown their advantages in middle-and long-range travel [8]. In the past 10 years, EBSSs have flourished around the world. EBSS operating companies have placed electric bicycles in crowded areas, such as residential communities, subway stations, and business districts, to provide short-and medium-range travel services to residents. The fee is based either on the usage time or on the distance covered.

EBSSs have gradually become popular across China since 2017. On 15 April 2019, the Department of Transportation issued a new national standard for electric bikes (e-bikes). The weight of the e-bike was limited to below 55 kg, and the speed was limited to below 25 km/h. The new national standard furthered the development and popularization of EBSS in China. In some large cities where motorcycles are forbidden and the infrastructure for non-motor vehicle transportation is relatively ideal, the EBSS is the best choice for residents to travel in urban area. In some small- or medium-sized cities, the EBSS has become the best choice for many citizens to commute because of the advantages of electric bicycles in medium-distance riding.

According to the China Bicycle Association, the production scale of electric bicycles in China reached 1 million in 2019. EBSSs were deployed in nearly 300 cities. As major cities in China accelerate their plan of EBSS deployment, it is expected that the EBSS market will exceed 8 million bicycles in 5 years, and the compound annual growth rate of the EBSS market will reach 41% [9].

User perception is considered to be one of the key issues to facilitate the orderly development of urban EBSSs. However, few research reports have analyzed EBSS user behavior. As a new type of travel service for urban residents, the EBSS includes the following: (1) a mobile app, which realizes the functions of user registration, pick-up, check out, payment, vehicle query, customer complaints and suggestions; (2) hardware, including electric bicycles, parking devices, and relevant smart sensors; and (3) a riding environment, including riding roads, traffic safety signs, and safety management specifications, to ensure smooth riding. Reliability of these elements will affect user perception. On the other hand, privacy protection, usage fees, and environmental protection values will also affect user participation.

The technology acceptance model (TAM) and expanded TAM have been widely used to explain the acceptance of new technologies and systems. There were no previous studies of EBSS user behavior perception based on the TAM framework. Based on this research gap, this research contributes to the acceptance theory by proposing a model (EBSS technology acceptance model, EBSS-TAM) that puts a spotlight on the influence of reliability, safety, cost, and environmental protection values on user intention to use EBSS services and on EBSS service acceptance.

The rest of this article is organized as follows. Section 2 surveys the literature related to EBSSs and the TAM. Section 3 proposes the conceptual model of the EBSS-TAM and clarifies our research hypotheses. Section 4 outlines the study design and statistical results. Finally, Section 5 discusses the conclusions and related management implications, as well as the limitations and prospects for future research.

## 2. Literature Review

### 2.1. Electric Bicycle and Electric Bicycle-Sharing Systems

(1) Electric bicycle.

Electric bicycles can be divided into two categories: bicycle-style electric bicycles and scooter-style electric bicycles. The latter are larger, heavier, and faster. There are many EBSSs around the world, such as the Bird in the USA and Europe, the Scoot Networks in the USA [6], and the MingGuo and the Youon in China. EBSS charge by usage time. For example, the cost for a Bird ride is USD 1 to start and USD 0.15 per minute fee to ride; renting a MingGuo electric scooter costs CNY 2 for the first 20 min and then CNY 1 per 10 min.

Compared with traditional bicycles, electric bicycles are faster, especially when riding uphill [10]. They can maintain speed with less effort and provide health benefits [11]. Riding electric bicycles can mitigate the effort to overcome some obstacles, such as hills and longer distances, and can also improve people’s mobility [12]. Over the last 20 years, the production rate of electric bicycles has increased considerably [13]. Just like the classic bicycle, electric bicycles are not troubled by many problems associated with petrol-fueled cars. Electric bicycles have better energy efficiency and emit fewer greenhouse gases than all other motorized vehicles [14,15]. Furthermore, as many health guidelines have begun to stress the importance of daily exercise, e-bikes outshine other means of transport by providing a chance for exercise [16].

(2) Electric bicycle-sharing systems.

EBSSs are public shared bicycle systems. As a supplement to private and public transportation, public shared bicycle systems (PSBS) are an indispensable part of urban transportation. Many scholars have researched PSBS operation management, and mainly focused on the following aspects:

(a) Station location. Lin et al. [17] formulated the problem as a joint hub location and inventory model, expressed it as an integer nonlinear programming problem, and solved this problem by CPLEX.
(b) Fleet dimensioning. Fleet dimensioning answers the following question: in given station locations, how many bicycles should be deployed to meet demand and thus ensure the system’s viability? Fricker and Gast [18] used queue theory to analyze and solve this problem.
(c) Station inventory. Station inventory refers to the optimization of the number of bicycles located at each station. Raviv and Kolka [19] investigated a station inventory problem in a bike-sharing environment. They modeled the problem as a dynamic inventory system and solved it by using heuristic algorithms.
(d) Bike-sharing rebalancing problem (BRP). In this problem, the objective is to minimize total cost of running a fleet of capacitated vehicles used for redistributing bikes. Ho and Szeto [20] tackled the static BRP with a hybrid large-neighborhood search. Several removal and insertion operators are proposed to diversify and intensify the search. A simple tabu search is further applied to the most promising solutions. Contardo et al. [21] proposed forward time-indexed formulations of dynamic BRPs and solved them via Dantzing–Wolfe and Benders’ decomposition heuristics.

Compared to PSBS research, relevant studies of EBSS operation management have rarely been reported. Cherry et al. [22] outlined the system requirements, operational concepts, and battery management of an innovative e-bike-sharing system. Chen et al. [23] studied the problem of how to deploy e-bike sharing stations and determine their capacities. The EBSS station deployment problem was then formulated as a bi-level programming model that took government and individual traveler profits into account. They used a hybrid particle swarm optimization algorithm to solve the model.

### 2.2. Usage Behavior Analysis of Electric Bike-Sharing Systems

Previous research on the usage behavior analysis of EBSSs focused on public perception [23,24], on analyses of user experiences [25,26], on evaluations of technological and environmental impacts [15,27], and on measures of the effects on health and safety [16,26]. Guanetti et al. [28] studied the energy management problem of electric bicycles. They tackled the problem using optimal control principles, built on an approximated solution of the problem, and proposed a control system that copes with incomplete trip information, aiming to minimize the perceived physical exertion while guaranteeing a predetermined electric range. Campbell et al. [29] employed a stated preference survey and multinomial logit model to analyze the factors influencing the choice to switch from an existing transportation mode to bikeshare or e-bikeshare in Beijing. They found that the bikeshare choice is most sensitive to measures of effort and comfort while the e-bikeshare choice is more sensitive to user heterogeneities. Kaplan [30] proposed a behavioral framework to examine the intentions of inhabitants in a driving-oriented region to use conventional electric bicycles within a hypothetical scenario involving a bicycle-sharing system. It was suggested that when promoting bicycle sharing, and more broadly, cycling, we should not only focus on the value as a means of transportation but also the social values and those in environmental protection and in self-actualization. Li et al. [31] extended the service quality model and theory of planned behavior (TPB) model with the residual effects of past behavior to explore the determinants of EBSS behavioral intention and behavior. It was found that shared electric bicycles’ service quality has a positive effect on people’s attitude toward shared electric bicycles and on behavior intention; the residual effects of past behavior has no effect on the further behavior of shared electric bicycles; perceived behavioral control positively affects shared electric user intention and positively moderates the relationship between attitude and behavioral intention. Li et al. [32] collected a sample of 751 respondents in a small-sized city in China to analyze residents’ intention to use shared electric bicycles and then used the extended TPB to analyze. They found that attitude, subjective norm, and perceived behavior control had direct positive effects on the user intention to use EBSS.

### 2.3. Technology Acceptance Model (TAM) and Its Extensions

The TAM, based on the TPB, was first introduced by Davis in 1989 [33]. It is widely used in explaining and predicting the usage of new technologies [34,35]. The main idea of this model is that there are two factors, perceived usefulness and perceived ease of use, which have a direct and indirect (via attitudes) effect on the intention to use a new technology. Perceived usefulness is defined as the prospective user’s subjective probability that using a specific application system will increase his or her job performance within an organizational context, and perceived ease of use is defined as the degree to which the prospective user expects the target system to be free of effort [34]. Davis et al. [36] also suggested that some external variables, such as quality of outcome, training, user support, and documentation, can affect perceived usefulness and perceived ease of use in some scenarios. Scholars have used the TAM to explain the user behavior of emerging technologies and information technologies, including DLBSSs. Kenneth and Yang [37] studied the factors that affected Singaporeans’ adoption of mobile commerce through the TAM model and tested their decision-making process for adopting mobile commerce by using regression analysis. Liu and Yang [38] integrated the subjective norm (SN) and imitating others (IMI) into the TAM to establish an extended TAM for applications of sharing economy, such as car sharing and bicycle sharing. Lyu and Zhang [39] proposed an extended model that integrated a TAM with DLBSS features to investigate user behavior of DLBSSs. The research results showed that the extended TAM model provided a more comprehensive understanding of DLBSS acceptance. Chen and Chao [40] examined whether perceived usefulness and perceived ease of use play a significant role in people’s decisions to choose public transportation. The results showed that both have positive and significant effects on attitudes towards using public transport.

The increasing number of new technologies makes it more difficult to predict consumers’ behaviors. Therefore, Venkatesh and Davis [41] added two groups of constructs (representing social influence and cognitive influence) that can be regarded as antecedents of perceived ease-of-use and of the intention to use new technologies into the classic TAM. They called this model TAM2. Subjective norm (SN) is one of the most important variables related to social influence in TAM2. It affects user intention via perceived usefulness in both direct and indirect ways.

Unified theory of acceptance and use of technology (UTAUT) is also an extension of TAM; it was proposed by Venkatesh et al. in 2003 [42]. UTAUT indicated that performance expectations, effort expectations, and social impact directly determined the behavior intentions, and the final behavior was determined by the behavior intentions and convenience conditions. Age, gender, and user experience were used as moderating variables to regulate the relationship among antecedent variables, behavior intentions, and behaviors. Scholars have performed a great deal of research to analyze users’ acceptance of new technology, willingness to use new technology, and behaviors related to new technology. For example, by using the UTAUT theory, Rodríguez and Carvajal [43] explored the factors that affect users’ behaviors when these users were booking flights from budget airline websites. Slade et al. [44] expanded the UTAUT theory by taking innovation, risk, and trust into consideration, and they also studied the determinants of consumers’ intentions of mobile payments in the UK.

UTAUT2 is an extension of UTAUT that retains all the core variables of the UTAUT theory and adds three variables: hedonic motivation, price value, and habit. Adjustment variables from the UTAUT model such as gender, age, and experience are retained in UTAUT2, but there is a sole exception—voluntary nature is excluded. Through a series of verifications of the UTAUT2 model, they found that the UTAUT2 theory has a 16% higher explanation degree of behavior intention and a 12% higher explanation degree of technology use than UTAUT theory did [45]. Based on the UTAUT2 theory, Ali et al. [46] selected students as the research objects to analyze the factors that affect a user’s willingness to use collaborative teaching; they used the partial least-squares method and verified that all variables had a significant impact on student’s willingness to use collaborative teaching. Oliveira et al. [47] studied determinants of mobile payment user behavior (adoption and recommendation) by combining the UTAUT2 theory with the innovation diffusion theory and applied their method in the field of mobile payments. Their results showed that mobile payment user behavior is affected by perceived technology security, innovation performance expectations, and compatibility.

The TAM and its extensions are now considered as the most influential models to explain how users accept and use various technologies. From these models, we found that perceived usefulness and ease of use may not fully explain users’ motives or attitudes. Some additional factors such as habits, enjoyment, motivation, attitudes, self-efficacy, social influence, and demographics may be associated with technology acceptance. We tried to integrate the following potential variables (perceived pleasure (PP), perceived environmental value (PEV), perceived cost (PC), and perceived reliability (PR)) into the traditional TAM. Unlike the reported research, PP and PEV are not subvariables of perceived usefulness (PU), but as latent variables are directly related to perceived ease of ues (PEU), behavior attitude (BA), and behavior intention (BI) in this model. PR and PC are treated as determinants of EBSS service in this model, and are related to BA as well as BI.

## 3. Theoretical Model and Hypotheses Development

**Figure 1:**
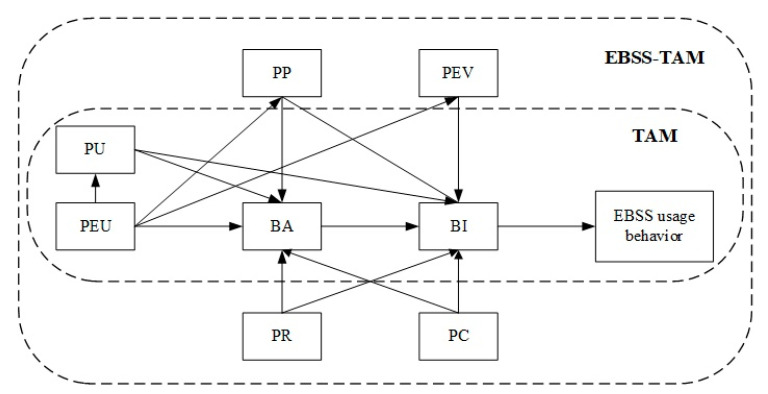
Conceptual model.

According to the TAM, the user behavior of the EBSS is influenced by perceived usefulness (PU), perceived ease of use (PEU), behavior attitude (BA), and behavior intention (BI). However, the EBSS is not just an IT system. From the perspective of a service provider, the EBSS is composed of a mobile app, e-bikes, app-compatible smart devices, and a riding environment. From the perspective of users, the value of the EBSS lies not only in realizing convenient urban travel but also in meeting the demand of social and environmental protection [15,27]. The EBSS is also considered as way to improve health and happiness [16,26]. Thus, by taking the theoretical background of the TAM and features of the EBSS into consideration, we proposed an EBSS technology acceptance model that integrates several latent variables that will be used as predictors of EBSS usage behavior. These variables include perceived pleasure (PP), perceived environmental value (PEV), perceived cost (PC), and perceived reliability (PR). The relationships among these variables are shown in Figure 1.

According to the classic TAM model, usage behavior is directly influenced by users’ behavior intention; users’ behavior intention is directly influenced by users’ behavior attitude and perceived usefulness; users’ behavior attitude is directly influenced by perceived usefulness and perceived ease of use; perceived usefulness is directly influenced by perceived ease of use. Research hypotheses H1–H6 are presented in Table 1.

(1) Perceived pleasure (PP). PP is a variable introduced in UTAUT2. It refers to the interest or pleasure obtained from the usage of a type of technology. In our model, PP refers to the degree of emotional pleasure that users think they can achieve by using an EBSS. Yi [29] pointed out that hedonic motivation has a direct impact on acceptance and on usage of technology. Jones et al. [16] investigated the determinants of e-bike rider experience and perceived impact. They found that e-bikes allowed participants to maintain physical activity, which benefits user health. This is an attractive feature that is not seen in traditional means of transportation. Thus, users may find it refreshing and entertaining, and enhance their willingness to use an EBSS. Research hypotheses H7–H9 are presented in Table 1.
(1) Perceived environmental value (PEV). Chen [48] defined the environmental value of DLBSSs. PEV can result in word-of-mouth promotion of DLBSSs and increase users’ reuse intentions. In our model, PEV is considered to be the degree to which individuals believe that using an EBSS could improve the environmental factors of certain aspects of their lives in an organizational context. The context includes efficiency, value, and productivity to address the value of self-care, transportation improvement, and green use. We propose hypotheses H10–H12 in Table 1.
(2) Perceived cost. The PC is a variable introduced in UTAUT2. It refers to the trade-off between the pay and return in the process of using the EBSS. Chan et al. [49] found that short message service (SMS) systems are very popular in China because the usage fees are relatively low. From a practical point of view, if the cost of shared transportation is lower than that of traditional transportation; users will have higher willingness to use it due to the benefits. We propose hypotheses H13–H14 in Table 1.
(3) Perceived reliability (PR). PR comes from the SERVQUAL model [50]. It is composed of five dimensions: tangibility, responsiveness, assurance, empathy, and reliability; PR has also been adapted in diverse service contexts. According to Shao et al. [51] the reliability of bicycle-sharing services is essentially related to the location reliability, transaction assurance, clean appearance, and fast service response. A safe riding experience and a user interface that is reliable and convenient may enhance a user’s willingness to use it. We propose hypotheses H15–H16 in Table 1.

**Table 1:** Research hypotheses.

ID	Hypotheses	Path
H1	Perceived usefulness has a positive effect on behavior attitude.	PU → BA
H2	Perceived usefulness has a positive effect on behavior intention.	PU → BI
H3	Perceived ease of use has a positive effect on behavior attitude.	PEU → BA
H4	Perceived ease of use has a positive effect on perceived usefulness.	PEU → PU
H5	Behavior attitude has a positive effect on behavior intention.	BA → BI
H6	Behavior intention has a positive effect on actual behavior.	BI → AB
H7	Perceived pleasure has a positive effect on behavior attitude.	PP→ BA
H8	Perceived pleasure has a positive effect on behavior intention.	PP → BI
H9	Perceived ease of use has a positive effect on perceived pleasure.	PEU → PP
H10	Perceived environmental value has a positive effect on behavior attitude.	PEV → BA
H11	Perceived environmental value has a positive effect on behavior intention.	PEV → BI
H12	Perceived ease of use has a positive effect on perceived environmental value.	PEU → PEV
H13	Perceived cost has a negative effect on behavior attitude.	PC → BA
H14	Perceived cost has a negative effect on behavior intention.	PC → BI
H15	Perceived reliability has a positive effect on behavior attitude.	PR → BA
H16	Perceived reliability has a positive effect on behavior intention.	PR → BI

## 4. Method

**Figure 2:**
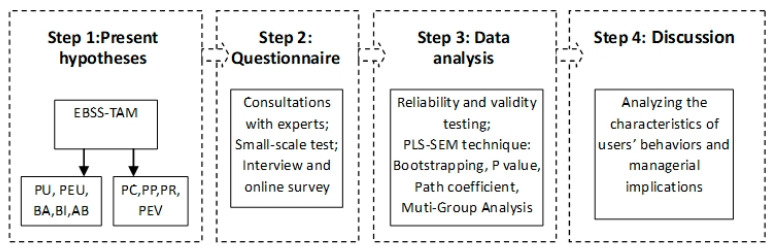
Study framework.

PU, PEU, PC, PP, PR, PEV, BA, BI, and AB are latent variables in our theoretical model. These variables could not be measured directly. Therefore, we designed observed variables to measure these latent variables indirectly, and these variables were confirmed by using some specific items. The measured values consisted of real values and measurement errors. However, by using the structural equation, we can considerably improve the accuracy of the overall measurement because structural equation eliminates random measurement errors that occur when analyzing the structural relationship among latent variables. The structural equation model (SEM) could be used to calculate relationships among multiple dependent variables concurrently, thus providing the most comprehensive and appropriate analysis when studying the mediating effect [52]. We adopted the partial least-squares algorithm (PLS) to examine the quality of structural models, and presented the overall framework of this study in Figure 2.

### 4.1. Questionnaire Design

We conducted a questionnaire survey to test our research hypotheses. The questionnaire consisted of three parts: questionnaire instructions, basic information of the respondent, and the EBSS-TAM scale. Based on consultations with experts and small-scale tests, the initial questionnaire was revised to form the final questionnaire. The basic information included gender, age, education, career, and user experience with CBSS. The EBSS-TAM scale consisted of nine latent variables. The latent variables were measured by using the Likert scales, and are summarized in Table 2.

### 4.2. Data Analysis

The questionnaires were delivered randomly to the EBSS’s users in the Yuetang District and Yuhu District of the city of Xiangtan. The city of Xiangtan is located in the center of Hunan Province. Yuetang District and Yuhu District are the main urban areas of Xiangtan. Together they have a total area of 657 square kilometers and a population of 1,070,000. The city area of Yuetang District and Yuhu District is relatively small, so the daily travel is mainly medium- and short-distance travel (3–10 km). As early as 2015, a unified public bicycle system with 10,000 bicycles and non-motor vehicle riding facilities was established in the main urban area; this system is operated by a state-owned enterprise. In June 2020, this enterprise began to deploy EBSSs in the main urban area. The utilization rate of EBSSs has been growing rapidly. So far, 15,000 e-bikes have been put into operation, while the previously deployed CBSS has been deactivated. Residents can utilize these shared electric bicycles in their daily travel. The problem of excessive motor vehicles in these areas has been significantly alleviated to a certain extent.

We issued paper-based questionnaires at schools, EBSS stations, and shopping centers during April and June in 2021, and most respondents were required to finish the questionnaires on site. The web-based questionnaires were sent by an online survey tool (accessed on 1 January 2020, www.wjx.cn). To encourage the respondents to complete the survey, we ensured that every respondent who successfully submitted the questionnaire was given “lucky money” as a gift through WeChat Wallet.

In this study, 436 questionnaires were collected, and of those questionnaires, 399 were valid. The statistics of gender, age, education level, occupation, and bike-sharing experience are shown in Table 3. Nearly half of the respondents were men, accounting for 48.1% of the respondents, 68.2% of the respondents were between the ages of 19–35, and 53.40% of respondents had bachelor’s degrees. The population statistics of gender and age were similar to those reported previously [32], but the occupation and bike-sharing system experience statistics showed regional characteristics. Reasons of these regional characteristics are as follows: Firstly, Xiangtan has more universities than other areas. Secondly, the BSS and EBSS are operated by the same enterprise in Xiangtan.

### 4.3. Measurement Model Assessment

The proposed research model has been tested through the PLS algorithm. We used Smart PLS 3.2.8 software to analyze the SEM indices. The long-tested PLS-SEM was used to test and evaluate complex statistical models and to deal with problems such as data noise or missing data [53]. We set the maximum iterations as 5000, and the PLS algorithm stopped when the change in the outer weights between two consecutive iterations was smaller than 10^−^^7^.

In this model estimation, factor loadings (λ), Cronbach’s α coefficient, composite reliability (CR), and average variance extracted (AVE) are commonly used to measure the reliability and convergent validity. When λ, Cronbach’s α, and CR values are greater than 0.7, the model is considered to be acceptable for internal consistency. In Table 4, the factor loadings (λ) are marked in bold, and all the values were greater than 0.723, which indicated that individual item reliability was acceptable.

Furthermore, AVE can be used to test differential validity, and in the reactive model, AVE should be at least greater than 0.5. As illustrated in Table 5, the values of Cronbach’s, CR and AVE of nine latent variables are 0.791 to 0.908, 0.905 to 0.932, and 0.713 to 0.859, respectively.

The discriminant validity is defined as the amount to which a set of items can distinguish a variable from other variables. In this model, we used the Fornell–Larcker criterion to detect the discriminant validity of latent variables, and used the heterotrait–monotrait (HTMT) ratio of correlations to confirm this test reliability. Cepeda Carrin et al. [54] highlighted that if the HTMT value is below 0.90, and discriminant validity has been established, then the model is reliable for further processing. In Table 6, the diagonal line is the discriminant validity, all the values are higher than 0.8, and each value is higher than other correlation values between latent variables. In Table 7, all HTMT ratios are lower than 0.90. We also use cross loading to test the discriminant validity of manifest variables; in Table 4, the factor loadings are significantly greater than cross loading. These indicated that the results obtained from the survey using the designed scale were reliable.

### 4.4. Structural Model and Hypothesis Testing

**Figure 3:**
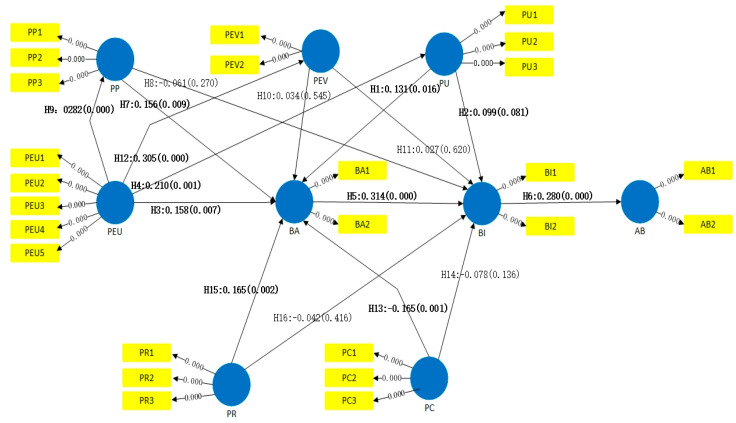
Structural model and path coefficients.

After establishing the outer model, the subsequent stage was to observe the proposed hypotheses. PLS-SEM employs bootstrapping to test the statistical significance of various PLS-SEM results. We used a bootstrap resampling procedure and set 5000 subsamples, and gave bootstrap standard errors, which in turn gave approximate T-values for significance testing of the structural path. The results of the bootstrapping procedure are shown in Table 8 and Table 9 and Figure 3. All the R-squared values ranged from 0.143 to 0.475. The higher the R-squared value, the greater the model’s predictive capacity for that variable, and the values must be greater than 0.10 with a significance of t > 1.64. All the Q-squared values ranged from 0.283 to 0.457, and the values must be greater than 0. Thus, cross-validated redundancy measures show that the theoretical/structural model has predictive relevance.

In our study, H1, H2, H3, H4, H5, H6, H7, H9, H12, H13, and H15 were supported (had significant effects), while H8, H10, H11, H14, and H16 were not supported (did not have significant effects). The path coefficients with significant influence are marked in bold in Figure 3.

Some interesting results were revealed in EBSS-TAM. (1) PP has a positive impact on BA, while having no impact on BI; (2) PEV has no impact on BA and BI; (3) PC has a negative impact on BA and has no impact on BI; (4) PR has a positive impact on BA while having no impact on BI; (5) PEU has a positive impact on PP and PEV.

### 4.5. Multigroup Analysis

The multigroup analysis (MGA) allows us to test if predefined data groups have significant differences in their group-specific parameter estimates. We selected four characteristic indicators for grouping: age (over 35/35 and under), gender (male/female), level of education (below undergraduate/undergraduate or above), and experience of using CBSS (never used/used). The MGA of SmartPLS, and setting 5000 subsamples of bootstrapping, were used to evaluate the structural model. Table 10 lists the paths with significant coefficient differences under different grouping conditions. All absolute values of path coefficient difference are greater than 0.23, and *p* values are less than 0.05 or greater than 0.95, indicating significant path coefficient differences between groups. The results of the multigroup analysis are as follows:

(1) Impact of behavior attitude on behavior intention: Both types of user behavior attitude have a significant positive impact on the behavior attitude, but the behavior attitudes of younger users (under 35 years old) were significantly stronger than those of older users.
(2) Impact of perceived ease of use on behavior attitude: Perceived ease of use is more likely to affect the user attitude of EBSSs on users with lower education background than on those with higher education background.
(3) Impact of perceived ease of use on perceived pleasure: Male users feel the pleasure brought by the ease of use of EBSSs more strongly than female users do.
(4) Impact of perceived ease of use on perceived environmental value: Both types of users’ perceived ease of use have a significant positive impact on perceived environmental value, but the perceived ease of use of the users who never used a CBSS brings a significantly stronger impact than that of users who used a CBSS before does.

**Table 10:** Multigroup analysis results.

Path	Coefficient								Absolute Value of Path Coefficient Difference	*p* Values	W-S Test (*p* Values)
	Over 35	35 and Under	Below Undergraduate	Undergraduate or Above	Never Used CBSS	CBSS Used	Female	Male			
BA → BI	0.121	0.417							0.293	0.996	0.010
PEU → BA			0.332	0.090					0.260	0.023	0.042
PEU → PEV					0.496	0.233			0.266	0.019	0.033
PEU → PP							0.176	0.405	0.231	0.023	0.045

## 5. Discussion and Managerial Implications

Based on the analysis results of the EBSS’s structural equation model, we can conclude that the classical TAM construct relationship is still valid in the EBSS context. BA has direct positive effects on BI; BI has direct positive effects on AB; PU has direct positive effects both on BA and BI; PEU has direct positive effects on BA and PU. The path coefficient from BA to AB is equal to the coefficient from BA to BI (0.325) multiplied by the path coefficient from BI to AB (0.278). The result is 0.090, indicating that there is a gap between the user attitude and behavior in EBSSs. Therefore, service providers should strive to improve the convenience of use of EBSSs by building bicycle lanes, developing quick pick-up and return devices, and issuing incentive measures to encourage users with positive attitude towards EBSSs.

In the EBSS-TAM, PP has direct positive effects on BA, but has no effects on BI. PEV has no effects on BA and BI. PEU has direct positive effects on PP and PEV. The results show that PP and PEV have no significant impact on users’ attitude and intention for EBSSs. Operations departments should put advertisements and invite users to try EBSSs for free, so the potential users may enjoy the fun of riding and understand the environmental protection value of the EBSS. PC has negative effects on BA, and PR has direct positive effects on BA, showing that riding cost and system reliability will affect user attitude, and that lower-cost, safe, and reliable EBSSs have a significant impact on users’ attitude.

The multigroup analysis results show that younger users (under 35 years old) are more likely to change from liking EBSSs to using EBSSs than older users are. Male users are more likely to be satisfied with EBSSs because of their ease of use. Therefore, we believe that young male users are the main target customers of EBSSs.

The multigroup analysis results also show that the users who never used CBSSs are more likely to perceive the environmental protection value of EBSSs from the easy-to-use features of EBSSs. We generally believe that the user experience with CBSSs has a significant impact on the use of EBSSs. Users who have never used CBSSs have high recognition of the environmental value of EBSSs. However, users with CBSS use experience will find that CBSSs are cleaner than EBSSs, because the riding process of CBSSs involves zero energy consumption and zero emissions. Therefore, in the next stage, public transportation authorities should pay attention to diverse user requirements when developing BSSs and optimally allocate human-riding, battery-driven, or hydrogen-driven shared bicycles to meet these diverse requirements.

## 6. Conclusions

We integrated the perceived reliability, perceived cost, perceived pleasure, and perceived environmental value into the original TAM model as latent variables and then established an EBSS-TAM to investigate the determinants of user behaviors of the EBSS. The research model can well explain users’ behaviors regarding the use of EBSS. There are two contributions in our study. First, we extended the application range of the TAM to the field of shared electric bicycles. Second, we used PLS-SEM to reveal the potential mechanism of how factors other than PU and PEU (such as perceived reliability, perceived pleasure, perceived environmental value and perceived cost) affect user behavior in the EBSS-TAM.

The theoretical and methodological limitations of this research provide insights for future research. First, we could examine the adoption of DLBSSs from the perspective of city authorities, major traffic participants, traditional bicycle suppliers, dealers, and even other competitors. Second, we could explore the possibility of incorporating the interactions of consumers, businesses, and their employees into the conceptual model in future work. Third, the area studied belongs to the small- and medium-sized city categories, and we can test whether conclusions of this article could be applied in large cities.

## Figures and Tables

**Table 2:** Measurement scale items.

Latent Variable	Observed Variables
Perceived usefulness (PU)	PU1. I think an EBSS is more useful than other means of transportation.
	PU2. I think an EBSS can improve the efficiency of travel.
	PU3. I think an EBSS is very useful for daily travel.
Perceived ease of use (PEU)	PEU1. I think it is very easy to learn to use shared electric bikes.
	PEU2. I think it is easy to use the e-bike app.
	PEU3. I think it is easy to rent out an e-bike.
	PEU4. I think it is easy to return an e-bike.
	PEU5. I think an e-bike is very simple to ride.
Perceived reliability (PR)	PR1. I think e-bike riding is very reliable.
	PR2. I think the traffic environment for e-bike riding is very safe.
	PR3. I do not think the EBSS platform will disclose users’ personal information.
Perceived environmental value (PEV)	PEV1. I think riding an e-bike can reduce carbon emissions.
	PEV2. I will choose a low-carbon and environmentally friendly way to travel.
Perceived pleasure (PP)	PP1. It makes me feel happy to use an EBSS.
	PP2. I think it is fun to travel with friends using an EBSS.
	PP3. I think traveling by an EBSS is good for my health.
Perceived cost (PC)	PC1. I think the fees of EBSS are very low.
	PC2. I think the deposits of EBSS are very low.
	PC3. I think using an EBSS can save travel expenses.
Actual behavior (AB)	AB1. I have used EBSS.
	AB2. I often use EBSS.
Behavioral intention (BI)	BI1. There is an EBSS nearby. I would like to use it.
	BI2. I would recommend that relatives and friends use EBSS.
Behavior attitude (BA)	BA1. I highly recommend EBSS.
	BA2. I think EBSS are very convenient.

**Table 3:** Demographic characteristics of the respondents.

Items	Type	Frequency	Percent
Gender	Male	192	48.1%
	Female	207	51.9%
Level of education	Master’s degree or above	47	11.8%
	Undergraduate	250	62.7%
	Junior college	58	14.5%
	High school	26	6.5%
	Junior high school and below	18	4.5%
Occupation	Student	88	22.1%
	Government agencies or institutions	74	18.5%
	Enterprise personnel	173	43.4%
	Freelance	64	16.0%
Age	Under 18	2	0.5%
	19–25	187	46.9%
	26–35	85	21.3%
	36–50	81	20.3%
	51 and above	44	11.0%
Have used CBSS	Never used	104	26.1%
	Often used	250	62.7%
	Occasionally used	45	11.3%

**Table 4:** Cross loading.

	AB	BA	BI	PC	PEV	PEU	PP	PR	PU
AB1	**0.925**	0.294	0.272	−0.090	0.024	0.078	0.102	−0.021	0.082
AB2	**0.893**	0.247	0.231	−0.010	0.021	0.092	0.090	−0.019	0.094
BA1	0.294	**0.928**	0.301	−0.111	0.185	0.238	0.257	0.228	0.216
BA2	0.260	**0.926**	0.323	−0.133	0.145	0.230	0.241	0.183	0.211
BI1	0.286	0.310	**0.929**	−0.110	0.087	0.109	−0.005	0.011	0.091
BI2	0.224	0.310	**0.914**	−0.096	0.102	0.147	0.115	0.045	0.187
PC1	−0.063	−0.066	−0.061	**0.854**	−0.002	0.113	0.054	0.047	−0.029
PC2	−0.073	−0.130	−0.107	**0.855**	−0.045	0.088	0.046	0.031	−0.024
PC3	−0.056	−0.141	−0.148	**0.915**	−0.002	0.073	−0.010	0.066	−0.057
PEV1	0.038	0.163	0.077	−0.009	**0.924**	0.306	0.222	0.069	0.282
PEV2	0.007	0.163	0.112	−0.010	**0.904**	0.249	0.190	0.189	0.231
PEU1	0.106	0.264	0.130	0.048	0.264	**0.894**	0.299	0.147	0.230
PEU2	0.058	0.170	0.120	0.067	0.251	**0.874**	0.235	0.155	0.177
PEU3	0.089	0.232	0.121	0.125	0.253	**0.867**	0.225	0.136	0.123
PEU4	0.071	0.134	0.045	0.181	0.237	**0.723**	0.150	0.187	0.114
PEU5	0.068	0.245	0.149	0.028	0.292	**0.886**	0.262	0.139	0.212
PP1	0.107	0.237	0.074	0.026	0.157	0.265	**0.891**	0.201	0.244
PP2	0.084	0.256	0.050	0.021	0.226	0.267	**0.907**	0.172	0.200
PP3	0.090	0.216	0.022	0.075	0.220	0.208	**0.850**	0.171	0.266
PR1	−0.024	0.186	0.033	−0.001	0.097	0.203	0.187	**0.867**	0.059
PR2	0.010	0.241	0.051	0.039	0.127	0.172	0.192	**0.898**	0.078
PR3	−0.074	0.158	−0.009	0.130	0.139	0.105	0.164	**0.805**	0.044
PU1	0.092	0.182	0.101	−0.044	0.266	0.182	0.244	0.089	**0.874**
PU2	0.096	0.199	0.130	−0.049	0.211	0.180	0.214	0.050	**0.893**
PU3	0.085	0.214	0.143	−0.027	0.226	0.196	0.214	0.036	**0.907**

**Table 5:** Measurement model evaluation.

Latent Variable	Cronbach’s Alpha	Composite Reliability (CR)	Average Variance Extracted (AVE)
AB	0.791	0.905	0.827
PU	0.902	0.932	0.774
PP	0.859	0.914	0.780
PR	0.869	0.908	0.713
PC	0.908	0.927	0.719
PEU	0.904	0.929	0.724
PEV	0.804	0.911	0.836
BA	0.836	0.924	0.859
BI	0.823	0.918	0.849

**Table 6:** Discriminant validity matrix (Fornell and Larcker criterion).

Latent Variable	AB	PU	PP	PS	PC	PEU	PEV	BA	BI
AB	0.909								
PU	0.096	0.880							
PP	0.106	0.265	0.883						
PR	−0.022	0.062	0.206	0.845					
PC	−0.059	−0.036	0.043	0.073	0.848				
PEU	0.093	0.208	0.282	0.175	0.095	0.851			
PEV	0.025	0.282	0.226	0.137	−0.01	0.305	0.914		
BA	0.299	0.231	0.269	0.222	−0.131	0.252	0.178	0.927	
BI	0.278	0.148	0.057	0.03	−0.112	0.138	0.102	0.336	0.921

**Table 7:** Discriminant validity matrix (heterotrait–monotrait ratio, HTMT).

Latent Variable	AB	PU	PP	PS	PC	PEU	PEV	BA
PU	0.115							
PP	0.128	0.305						
PR	0.050	0.071	0.234					
PC	0.075	0.043	0.08	0.106				
PEU	0.11	0.223	0.309	0.193	0.121			
PEV	0.03	0.33	0.273	0.168	0.027	0.356		
BA	0.365	0.265	0.315	0.249	0.132	0.283	0.217	
BI	0.341	0.174	0.094	0.046	0.108	0.155	0.127	0.406

**Table 8:** Structural model results.

	R^2^	Sample Mean	Standard Deviation	T Statistics	*p* Values	Q^2^
AB	0.277	0.182	0.070	3.980	0.009	0.398
BA	0.475	0.194	0.069	6.898	0.000	0.457
BI	0.328	0.143	0.066	4.985	0.000	0.438
PEV	0.393	0.299	0.068	5.815	0.000	0.413
PP	0.279	0.284	0.053	5.282	0.000	0.311
PU	0.143	0.248	0.056	2.548	0.010	0.283

**Table 9:** Hypotheses testing results.

Hypotheses	Path	Coefficient	T Statistics	*p* Values	Testing Results
H1	PU → BA	0.131	2.405	0.016	Supported
H2	PU → BI	0.099	1.748	0.081	Supported
H3	PEU → BA	0.158	2.686	0.007	Supported
H4	PEU → PU	0.210	3.414	0.001	Supported
H5	BA → BI	0.314	5.824	0.000	Supported
H6	BI → AB	0.280	5.192	0.000	Supported
H7	PP → BA	0.156	2.611	0.009	Supported
H8	PP → BI	−0.061	1.103	0.270	Not supported
H9	PEU → PP	0.284	4.858	0.000	Supported
H10	PEV → BA	0.034	0.606	0.545	Not supported
H11	PEV → BI	0.027	0.496	0.620	Not supported
H12	PEU → PEV	0.306	5.056	0.000	Supported
H13	PC → BA	−0.165	3.312	0.001	Supported
H14	PC → BI	−0.078	1.491	0.136	Not supported
H15	PR → BA	0.165	3.065	0.002	Supported
H16	PR → BI	−0.042	0.813	0.416	Not supported

Note: Testing results are at significance level 0.1.

## Data Availability

The study does not require the public reporting of data.
